# Expression of decitabine-targeted oncogenes in meningiomas in vivo

**DOI:** 10.1007/s10143-022-01789-1

**Published:** 2022-04-21

**Authors:** Julian Canisius, Andrea Wagner, Eva Christina Bunk, Dorothee Cäcilia Spille, Louise Stögbauer, Oliver Grauer, Katharina Hess, Christian Thomas, Werner Paulus, Walter Stummer, Volker Senner, Benjamin Brokinkel

**Affiliations:** 1 Department of Neurosurgery, University Hospital Münster, North Rhine Westphalia, Albert-Schweitzer-Campus 1, Building A1, 48149 Münster, Germany; 2 Institute of Neuropathology, University Hospital Münster, Münster, North Rhine Westphalia Germany; 3 Department of Neurology With Institute of Translational Neurology, University Hospital Münster, North Rhine-Westphalia, Münster, Germany; 4 Department of Pathology, University Hospital Schleswig-Holstein, Campus Kiel, Kiel, Germany

**Keywords:** Chemotherapy, Decitabine, ELOVL2, FAM84B, Meningiomas, TRIM58

## Abstract

Treatment of meningiomas refractory to surgery and irradiation is challenging and effective chemotherapies are still lacking. Recently, in vitro analyses revealed decitabine (DCT, 5-aza-2’–deoxycytidine) to be effective in high-grade meningiomas and, moreover, to induce hypomethylation of distinct oncogenes only sparsely described in meningiomas in vivo yet.

Expression of the corresponding onco- and tumor suppressor genes *TRIM58*, *FAM84B*, *ELOVL2*, *MAL2*, *LMO3*, and *DIO3* were analyzed and scored by immunohistochemical staining and RT-PCR in samples of 111 meningioma patients. Correlations with clinical and histological variables and prognosis were analyzed in uni- and multivariate analyses.

All analyzed oncogenes were highly expressed in meningiomas. Expression scores of *TRIM58* tended to be higher in benign than in high-grade tumors 20 vs 16 (*p* = .002) and all 9 samples lacking *TRIM58* expression displayed WHO grade II/III histology. In contrast, median expression scores for both *FAM84B* (6 vs 4, *p* ≤ .001) and *ELOVL2* (9 vs 6, *p* < .001) were increased in high-grade as compared to benign meningiomas. *DIO3* expression was distinctly higher in all analyzed samples as compared to the reference decitabine-resistant Ben-Men 1 cell line. Increased *ELOVL2* expression (score ≥ 8) correlated with tumor relapse in both uni- (HR: 2.42, 95%CI 1.18–4.94; *p* = .015) and multivariate (HR: 2.09, 95%CI 1.01–4.44; *p* = .046) analyses.

All oncogenes involved in DCT efficacy in vitro are also widely expressed in vivo, and expression is partially associated with histology and prognosis. These results strongly encourage further analyses of DCT efficiency in meningiomas in vitro and in situ.

**Supplementary Information:**

The online version contains supplementary material available at 10.1007/s10143-022-01789-1.

## Introduction

Meningiomas are the most common primary intracranial neoplasms and are usually treated by microsurgical resection and/or radiation therapy. Although the vast majority of meningiomas are characterized by benign biological behavior and therefore correspond to WHO grade I, about 20% of the tumors display frequent recurrences (50–90%) and increased mortality and are therefore classified as grade II and III lesions [11, 27]. Aside from the WHO grade of the tumor, the extent of tumor resection according to the Simpson classification system and, more significantly, the volume of the tumor left behind after microsurgery have been shown to correlate strongly with the risk of tumor recurrence [31, 32]. In fact, proximity to critical neurovascular structures and invasion of the adjacent bone and soft tissue can distinctly alter the extent of tumor resection and therefore directly impact the risk of postoperative tumor relapse. Hence, treatment of tumors refractory to microsurgery and irradiation is a key challenge during neuro-oncological care for meningioma patients, and further, e.g., chemotherapeutical options are urgently needed.

Over the last decades, a number of substances including cyclophosphamide-doxorubicin-vincristine chemotherapy, antiangiogenic drugs (e.g., bevacizumab), and tyrosine kinase inhibitors such as vatalanib, sunitinib, or trabectedin have been investigated in the treatment of meningiomas in mostly small and retrospective series, and only displayed limited effects on tumor control [11]. The efficacy of checkpoint inhibitors such as pembrolizumab (NCT03279692) or nivolumab (NCT02648997, NCT03173950) remains to be determined in currently ongoing clinical trials.

Decitabine (DCT, 5-aza-2’–deoxycytidine) is a demethylating agent commonly used in the treatment of hematopoietic malignancies (e.g., acute myeloid leukemia [16]) and also induces demethylation in soft tissue tumors such as sarcoma, which display both genetic and morphological similarities to meningiomas [3, 15, 19]. Recent in vitro analyses revealed a dose-dependent efficiency of DCT also in high-grade meningiomas, with specific DNA demethylation of several onco- or tumor suppressor genes (*TRIM58*, *FAM84B*, *ELOVL2*, *MAL2*, *LMO3*, *DIO3*), which have been hardly investigated in meningiomas yet [33]. Regarding the promising results from these in vitro analyses, we therefore (i) investigated the expression of the onco- and tumor suppressor genes differentially methylated by DCT in meningiomas in vivo and (ii) analyzed correlations with clinical and histological variables and prognosis.

## Materials and methods

### Patient selection

Clinical, radiological, and histological data were obtained from the institutional meningioma database, containing information of 1302 surgeries performed in our department between 1991 and 2018. For this study, 111 patients who underwent surgery for primary diagnosed intracranial grade I (*N* = 54, 49%) and II/III (*N* = 57, 51%) meningioma with complete information on age, sex, tumor location, extent of resection, and with a postoperative follow-up period of at least 60 months were selected. The collective was intendedly enriched for high-grade lesions to enable expression analyses in a sufficient number of patients.

### Clinical, radiological, and histological data recovery

Clinical, radiological, and histological data recovery has been described previously in detail. Briefly, archives of the local Institute of Neuropathology were reviewed for all histopathologically confirmed meningiomas resected in our department between 1991 and 2018. Meningioma subtype and histopathological grading were diagnosed according to the current 2016 WHO criteria. Hence, brain invasion was diagnosed in case of “irregular, tongue-like protrusions of tumor cells infiltrating underlying parenchyma, without an intervening layer of leptomeninges” on hematoxylin and eosin and Elastica van Gieson-stained slides. Maximum safely achievable tumor resection was performed in all patients and intraoperatively quantified according to the Simpson classification, as it is standard in our institution. Adjuvant irradiation was administered for primary diagnosed grade III and recurrent or subtotally resected grade II tumors as well as in benign lesions following simple debulking. Preoperative physical state was quantified according to the Karnofsky Performance Score (KPS). Tumor location was obtained from preoperative imaging and classified as described below.

Patients were followed-up by magnetic resonance imaging (MRI) and physical examination 3 months after surgery. Contrast-enhanced CT scans were performed in case of any contraindications for MRI, and imaging and examinations were repeated in 12- and 6-month intervals for benign and high-grade meningiomas, respectively. After 5 years of an event-free course, follow-up was repeated in bi-annual and annual intervals in grade I and II/III lesions, respectively. Imaging was evaluated by a team of two independent observers (at least one neurosurgeon and one (neuro-)radiologist) and progression or recurrence was diagnosed in case of any detected tumor growth, independent of the indication for subsequent therapy. Progression-free interval (PFI) was calculated from the date of surgery to the date of radiologically confirmed tumor progression or, in case of an event-free follow-up, to the date of the last follow-up.

### Expression analyses—immunohistochemistry

Expression of *TRIM58*, *FAM84B*, *ELOVL2*, *MAL2*, and *LMO3* was analyzed by immunohistochemical staining. Hence, formalin-fixed, paraffin-embedded Sects. (3–4 µm) were deparaffinized and rehydrated through a graded alcohol series according to standard protocols. Antigens were retrieved in sodium citrate buffer (pH = 6.0 Target Retrieval Solution S2369 DAKO; Agilent Technologies, Inc., Santa Clara, CA, USA, 1:10 diluted in distilled water) by the method of heat activation (40-min steam cooker, 20 min cooled by room temperature). Staining was performed using an Agilent Autostainer Link 48 with the DCS DetectionLine (CEA1706) Kit. The sections were stained with DAB (3,3-diaminobenzidine)-Chromogene (DC135C006 DCS) and then counterstained with hematoxylin, then dehydrated in an ascending series of alcohols (70%,96%,99%, Xylol), and finally sealed with Eukit and a coverslip for microscopic evaluation (Olympus BX-51). Table 1 specifies the applied antibodies and dilutions. Expression was analyzed by a team of two independent observers (JC and CT) and, for statistical reasons, quantified according to scores by two independent observers as follows. As technical negative controls, staining was performed without the primary antibody, while vascular endothelium served as biological negative controls in all cases. For quantification of oncogene expression, previously published scoring systems were chosen whenever available.

**Table 1:** Summarization of the used antibodies. The table reveals the analyzed antigen, the manufacturer of the antibody with the corresponding order number, the applied dilution for staining, and the host species

Antigen	Manufacturer	Order#	Dilution	Host species
FAM84B	OriGene Technologies, Rockville, MD, USA	TA501992	1:150	mouse
TRIM58	Sigma Aldrich, Merck KGaA, Darmstadt, Germany	HPA023637	1:200	rabbit
MAL2	Abcam, Cambridge, UK	ab217919	1:100	rabbit
LMO3	Abcam, Cambridge, UK	ab230490	1:100	rabbit
ELOVL2	Thermofisher Scientific, Schwerte, Germany	PA5-26,199	1:100	rabbit

For *TRIM58*, staining intensity was classified as 0 points (negative), 4 points (weak intensity), 8 points (moderate intensity), or 12 points (strong intensity). Staining density was classified based on the percentage of cells stained as 0 points (0%), 4 points (1–25%), 8 points (26–50%), 10 points (51–75%), or 12 points (> 75%). The final score was calculated as the sum of the intensity score and the density score as reported previously [23]. *MAL2* expression was dichotomously registered as absent (< 5% of immunopositive tumor cells) or present in five separate 400 × high-power microscopic fields. Expression intensity of *FAM84B* was quantified as 0 (negative), 1 (weakly positive), 2 (moderately positive), and 3 (strongly positive), and the percentage of positive cells was classified as 0 (negative–10%), 1 (11–25%), 2 (25–50%), and 3 (> 50%). The final score was then calculated by multiplication of the staining intensity with the percentage of positive staining cells according to previous descriptions [37]. For *LMO3*, staining intensity was quantified as absent (1), weak (2), moderate (3), or strong (4), and the percent of membranous and cytoplasmic staining in tumor cells was classified as 1 (0–25%), 2 (26–50%), 3 (51–75%), and 4 (76–100%). The final score was calculated by multiplication of the staining intensity with density [24]. For *ELOVL2*, staining intensity was evaluated in absent (0), weak (1), moderate (2), and strong (3), as the percentage of positively stained cells was subdivided in 1 (1–25%), 2 (26–50%), 3 (50–75%), and 4 (76–100%). The final score was calculated by multiplication of staining intensity and percentage of positively stained cells.

### Expression analyses—qRT-PCR

As a reliable antibody against *DIO3* was not available, we performed quantitative real-time PCR. Fifteen frozen samples (10 × grade I, 5 × grade II/II) were used for RNA extraction (Maxwell 16 simplyRNA Tissue Kit, Promega). QRT-PCR was performed using commercial TaqMan Assays (DIO3: Hs00956431_s1 and GAPDH: Hs02786624_g1 as housekeeping gene) in a StepOne Plus (Applied Biosystems) as described by the manufacturer. Results were normalized to the *DIO3* expression of the meningioma cell line Ben-Men-1, that was set to 1.

### Statistical analyses

All calculations were performed using standard commercial statistic software (IBM SPSS Statistics, Version 28, IBM, Germany). Data are described by standard statistics with median and range for continuous and absolute and relative frequencies for categorical variables. For statistical reasons, the tumor location was classified as “convexity/parasagittal” vs “skull base”. Similarly, the extent of resection was dichotomously registered as gross (GTR, Simpson grades I–III) and subtotal resection (STR, Simpson grades IV and V). In univariate analyses, correlations between categorical and continuous variables were investigated by Fisher’s exact and Mann–Whitney-*U* tests, respectively. Distribution of PFS was visualized by Kaplan–Meier plots and compared by Log-rank tests. Multivariate analyses were performed using the Mantel-Cox test and backward Wald logistic regression and characterized by hazard (HR), 95%-confidence intervals (CI), and Wald-test *p*-values. The following variables were tested in multivariate regression models (ref = reference): age, sex (male (ref) vs. female), WHO-grade (classified into grade I (ref) vs. high-grade, II/III), tumor location (classified as described above, “convexity/parasagittal” = ref), and degree of resection (classified into GTR (ref) vs. STR). A *p*-value of < 0.05 was considered statistically significant throughout the whole analyses. All reported *p*-values are two-sided. Data collection and scientific use were approved by the local ethics committee and approved by the patients in each single case (Münster 2007–420-f-S and Münster 2018–061-f-S).

## Results

### Clinical and histological characteristics

Table 2 summarizes baseline clinical, radiological, and histopathological data. As expected, the extent of resection strongly correlated with tumor location, and GTR was more commonly achieved in convexity/parasagittal than in skull base tumors (*N* = 48 of 59, 81% vs *N* = 30 of 52, 57%; *p* = 0.007). Within a median follow-up of 79 months (mean: 109 months, range: 60–284 months), tumor recurrence was observed in 44 cases (40%) and occurred in 32 of 57 high-grade but in 12 of 54 benign meningiomas (56% vs 22%, *p* < 0.001). No correlations between the extent of resection (*p* = 0.210) or tumor location (*p* = 0.083) and recurrence were found. Multivariate analyses adjusted for patients’ age, sex, tumor location, and extent of resection confirmed high-grade histology as the only independent predictor of tumor recurrence (HR: 2.30, 95%CI 1.17–4.52; *p* = 0.016).

**Table 2:** Baseline clinical and histopathological data. Information was available in almost all included patients (*KPS*, Karnofsky Performance Score)

Variable	*N* (*n*%)	Available data (*n*%)
Age (median, range; years)	58, 18–82	111 (100%)
Sex		111 (100%)
Females	65 (59%)	
Males	46 (41%)	
KPS (mean, range)	80 (50–100)	109 (98%)
Tumor location		111 (100%)
Convexity/parasagittal	59 (53%)	
Skull base	52 (47%)	
Extent of resection		
Histology		111 (100%)
WHO grade I	54 (49%)	
WHO grade II/III	57 (51%)	
Adjuvant irradiation	39 (35%)	108 (97%)

### Oncogene expression and correlation with clinical and histological variables

**Fig. 1:**
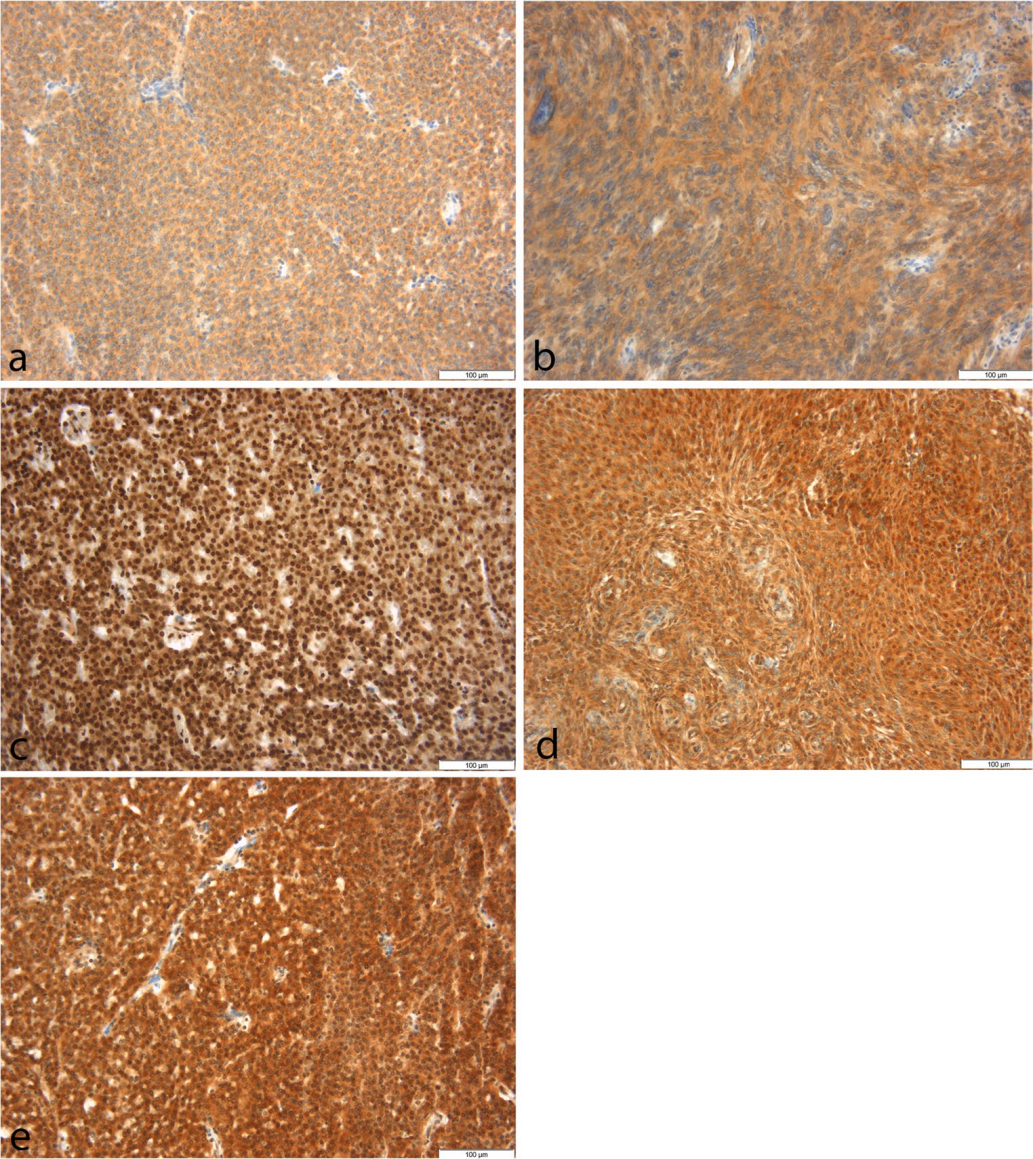
Representative images from immunohistochemical staining. Expression of the analyzed oncogenes *TRIM58* (**a**), *FAM84B* (**b**), *ELOVL2* (**c**), *MAL2* (**d**), and *LMO3* (**e**) was detected in most tumors with variable density and intensity. For illustration, samples with strong expression of all onco-/tumor suppressor genes were selected (magnification 200-fold, corresponding antibodies summarized in Table 1)

Immunohistochemical staining revealed a distinct expression of all analyzed oncogenes in the majority of tumor samples (Fig. 1). On visual inspection, expression was cytoplasmic in *TRIM58* and *FAM84B* and detected both in the nucleus and the cytoplasm in *MAL2* and *ELOVL2* and *LMO3*. Correlations between expression scores and clinical variables were mostly lacking and are summarized in Table 3. However, distinct relations between histology and expression were found.

**Table 3:** Correlation of expression of TRIM58, FAM84B, ELOVL, or MAL2 with clinical variables (*p*-values). Although reaching level of statistical significance, median *FAM84B* expression was only slightly higher in males than in females (6, range 1–9 vs 4, range 0–9; *p* = .021). No further correlations were found

Variable	TRIM58	FAM84B	ELOVL2	MAL2	LMO3	DIO 3
Age (median, range; years)	.657	.962	.643	.921	n.a.^a^	n.a
Sex	.616	.009	.969	.519	n.a	n.a
Tumor location	.413	.056	.571	.580	n.a	n.a

^a^Not applicable due to the low sample size

**Fig. 2:**
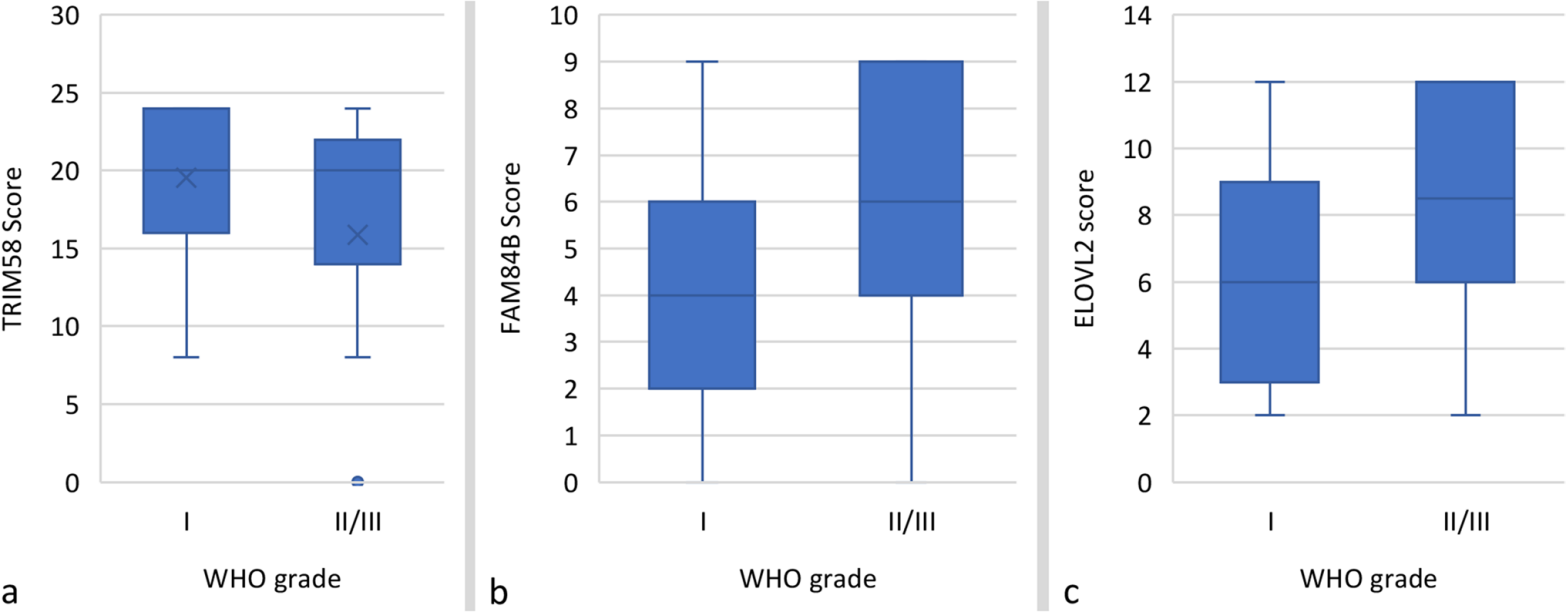
Box and whisker plots illustrating correlations of the *TRIM58*, *FAM84B*, and *ELOVL2* expression with histology. Although median expression score was 20 in both groups, the Kruskal–Wallis test and the corresponding plot were suggestive for higher *TRIM58* expression levels in grade I as compared to grade II/III meningiomas (*p* = .034, **A**), and *mean* expression score higher in grade I than in high-grade lesions (20 vs 16, *p* = .002, indicated with x). In contrast, both median *FAM84B* (6, range 0–9 vs 4, range 0–9; *p* ≤ .001, **B**) and *ELOVL2* (9, range: 2–12 vs 6, range: 2–12; *p* < .001, **C**). Expression scores were higher in grade II/III than in benign meningiomas. The boxes indicate upper and lower 25% quartile, the whiskers the minimum and maximum value, the dots the outliers, the asterisks the extreme values, and the heavy horizontal line indicates the median

In 101 samples successfully stained for *TRIM58* (91%), the median expression score was 20 and ranged from 0 (*N* = 9) to 24 (*N* = 19). Although median expression scores were 20 in both high-grade and benign meningiomas, the Kruskal–Wallis test revealed a statistically significant correlation between histology and *TRIM58* expression (*p* = 0.034, Fig. 2A). In fact, *mean* expression score was 20 (SD ± 4) in benign and 16 (± 8) in high-grade meningiomas (*p* = 0.002). Correspondingly, all 9 samples lacking *TRIM58* expression displayed high-grade histology. Immunohistochemistry for *FAM84B* was successful in 97 cases (87%) and displayed a median expression score of 6 (range: 0–9). Expression was slightly higher in males than in females (mean score 6, range 1–9, vs 4, range: 0–9; *p* = 0.009). Moreover, median *FAM84B* expression scores were increased in high-grade (6, range 0–9) as compared to WHO grade I meningiomas (4, range 0–9; *p* ≤ 0.001, Fig. 2B). Similarly, immunohistochemistry for *ELOVL2* showed expression in all analyzed samples (*N* = 103) with a median score of 8 (range: 2–12), and expression was higher in grade II/III (9, range: 2–12) than in grade I tumors (6, range 2–12; *p* < 0.001, Fig. 2C).

As previous reports already demonstrated expression in meningiomas and correlation with histology by microarray analyses and gene arrays, immunohistochemical slides for *MAL2* [10] and *LMO3* [30] were subjected to interim analyses. For *MAL2*, all 52 analyzed cases including 32 benign and 20 high-grade meningiomas displayed immunopositivity with strong expression (median 6, range 1–12) in most (N = 45) cases. In samples from six grade I and five grade II/III meningiomas subjected to *LMO3* immunohistochemistry, expression was strong in all samples (median score 12, range 4–16) and no further staining was performed.

qRT-PCR showed a median relative expression of *DIO3* of 140.15 (range: 3.37–10,286.51), which was distinctly higher as compared to the decitabine-resistant reference cell line Ben-Men 1, in all samples (suppl Fig. 1). Statistical analyses revealed a brought range but similar median expression values in (*N* = 9) grade I as compared to (*N* = 6) high-grade meningiomas (140.15, range 3.38–3572.39 vs 263.56, range: 9.65–10,286.51; *p* = 0.556).

### Correlation of oncogenes with recurrence

**Fig. 3:**
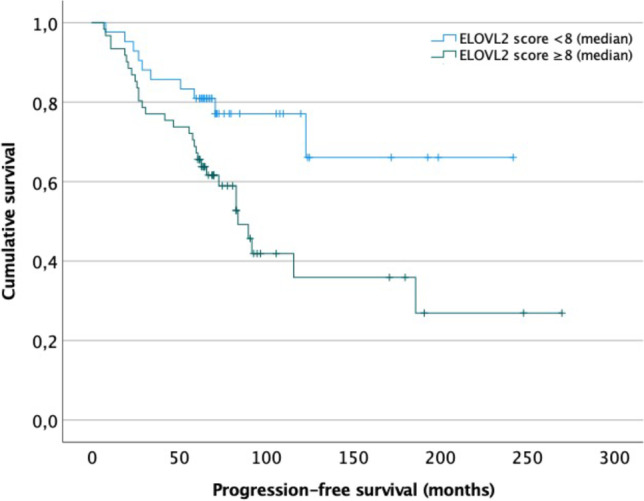
Correlation between progression-free survival and expression of *TRIM58* and *ELOVL2*. Increased expression of *ELOVL2* was correlated with shorter progression-free survival (*p* = .012, Log-rank test)

Correlations between recurrence and *TRIM58*, *FAM84B*, and *ELOVL2* expression were analyzed in univariate analyses as well as in multivariate tests adjusted for age, sex, tumor location, extent of resection, and, most notably, histology (Table 4). For statistical reasons, expression scores were dichotomized into < vs ≥ median score of each oncogene. Here, an increased *ELOVL2* expression (score ≥ 8) was identified as a strong risk factor for tumor relapse in both uni- (HR: 2.42, 95%CI 1.18–4.94; *p* = 0.015) and multivariate (HR: 2.09, 95%CI 1.01–4.44; *p* = 0.046) analyses (Fig. 3). *TRIM58* expression tended to correlate with recurrence in multi- (HR: 1.86, 95%CI 1.00–3.52; *p* = 0.056) but not in univariate analyses (HR: 1.74, 95%CI 0.92–3.29; *p* = 0.086), but without reaching the level of statistical significance. No further correlations between prognosis and the analyzed oncogenes were found.

**Table 4:** Correlations between the analyzed oncogenes and recurrence. *ELOVL2* expression above median was found a strong risk factor for tumor relapse in both uni- and multivariate analyses

	Univariate analyses		Multivariate analyses^b^	
Oncogene expression^a^	HR, 95%CI	*p*-value	HR, 95%CI	*p*-value
TRIM58	1.74, .92–3.29	.086	1.86, 1.00–3.52	.056
FAM84B	1.23, .65–2.33	.524	.85, .43–1.70	.645
ELOVL2	2.42, 1.18–4.94	.015	2.09. 1.01–4.33	.046
MAL2	1.79, .65–4.92	.262	1.35, .47–3.83	.579

^a^Expression scores were dichotomized into < (reference) vs ≥ median score of each oncogene

^b^Adjusted for patients’ age, sex, tumor location, extent of resection, and histology

## Discussion

The treatment of meningiomas refractory to surgery and/or irradiation remains challenging during neuro-oncological care. Recently updated treatment guidelines again reported limited efficacy of chemotherapy, hence underlining the urgent need for further laboratory and clinical research [12].

DCT is a DNA methyl transferase-inhibitor commonly applied in the treatment of AML. In vitro analyses have also shown efficiency in further malignancies and, noteworthy, a tumor-specific DNA demethylation [14]. In meningiomas, we could recently demonstrate a distinct and dose-dependent reduction of viability and proliferation in malignant, but not in benign meningioma cell lines following exposition to DCT. Remarkably, genome-wide DNA methylation analyses following drug exposition showed a specific DNA demethylation in DCT-sensitive, but not in DCT-refractory meningioma cells, hence suggesting molecular alterations underlying DCT sensitivity. In fact, demethylated regions included promoter regions of six tumor suppressor/ oncogenes (*TRIM58*, *FAM84B*, *ELOVL2*, *MAL2*, *LMO3*, *DIO3*) [33], whose expression in vivo is further characterized in the present study.

Expression of *TRIM58* was found in the majority of analyzed meningioma samples. *TRIM58* belongs to the tripartite motif protein (TRIM) family of E3 ubiquitin ligases and is considered a candidate tumor suppressor. Aberrant gene methylation of *TRIM58* has been shown in several malignancies including liver [28], lung, and colorectal cancer, induces silencing [18], and is associated with poor prognosis [23]. Correspondingly, expression in our study was higher in grade I than in grade II/III tumors and the only tumors lacking *TRIM58* immunopositivity were grade II/III lesions. Correlations with recurrence were rather ambiguous and lacking statistical significance.

*FAM84B* (Family With Sequence Similarity 84 Member B) is considered an oncogene which has been described in pancreatic [37], gastric [38], prostate [35], and esophageal [6] cancer, and has also been shown to correlate with tumor progression [6, 35]. In our study, *FAM84B* expression was found in all meningioma samples and was increased in high-grade as compared to benign tumors. However, no correlation with prognosis was found.

*ELOVL2* (Elongation Of Very Long Chain Fatty Acids Protein 2) is widely considered a biomarker for aging and silencing by DNA methylation [5, 9] and has been occasionally described in the context of oncogenesis, e.g., in breast and renal cell cancer or neuroblastoma [8, 17, 34]. Remarkably, *ELOVL2* has been proposed as both a tumor suppressor [8, 17] and, vice versa, a proto-oncogene [34]. As the first study so far, we revealed a brisk *ELOVL2* expression in meningiomas, which was additionally increased in high-grade as compared to benign lesions. *ELOVL2* expression was also associated with a > twofold risk of tumor relapse independent of the WHO grade of the tumor.

*DIO3* (Iodothyronine Deiodinase 3) plays a major role during embryogenesis and has also been described to promote cancer development by inhibiting tumor-suppressive actions of thyroid hormone T3 in several malignancies such as ovarian [26], lung [25], or prostate [13] cancer. Given the lack of a sufficient antibody for immunohistochemistry, we analyzed the expression in our series by RT-PCR. Here, normalized for DCT-resistant Ben-Men 1 cells, *DIO3* expression was distinctly increased in all samples but independent of the WHO grade.

*LMO3 (LIM domain only protein 3*) is a transcription co-factor interacting with p53 [20] and considered an oncogene, e.g., in neuroblastoma [1]. *MAL2* (Mal, T Cell Differentiation Protein 2), a transmembrane protein of the MAL proteolipid family, is upregulated in a number of malignancies, such as breast, colorectal, pancreatic, or ovarian cancer, has been shown to correlate with invasion and worse prognosis [2, 4, 22, 36]. In meningiomas, three previous studies analyzed *MAL2* and *LMO3* expression. In contrast to studies reporting the promotion of invasion and proliferation by *LMO3* in gastric and hepatocellular carcinoma [7, 29], Serna et al. showed a decreased expression in biologically aggressive meningiomas [30]. Our series basically confirmed the brisk expression of *LMO3* in meningiomas, while immunopositivity hardly varied in exploratory analyses and correlations with histology or prognosis were not investigated. For *MAL2*, noteworthy, previous studies reported promotor hypermethylation and downregulation in high-grade and recurrent meningiomas [10, 21]. Our study confirmed a brisk expression, while correlations with prognosis or histology were not found.

The authors are aware of some limitations of the study. The small sample size limits transferability and may lead to selection bias. Although clinically and histopathologically well-characterized, molecular information such as TERT promotor mutation status or DNA methylation classes of the patient collective were not available. Due to methodology, immunohistochemical staining only enables semi-quantitative analyses. Hence, while providing important exploratory analyses, determination of the role of the reported oncogenes for tumorigenesis in meningiomas remains to be further determined, e.g., by RNA sequencing or knock-out models.

In conclusion, this is the first study reporting the expression of the oncogenes *TRIM58*, *FAM84B*, *ELOVL2*, and *DIO3* in meningiomas and correlations with histology and prognosis. Moreover, we confirmed a brisk expression of *MAL2* and *LMO3*, underlining the importance during tumorigenesis. Previous molecular analyses already revealed the regulation of some of these oncogenes by DNA hypermethylation. Hence, these results further explain the efficiency of DCT in high-grade meningiomas in vitro and strongly encourage future in vitro and, potentially, in situ investigations.

## Supplementary Information

Below is the link to the electronic supplementary material.

Supplementary file1 Suppl. Fig1 qRT-PCR data of DIO3 expression in meningiomas. DIO3-RNA was expressed in all investigated frozen samples to a distinctly higher level as compared to the DCT-resistant meningioma reference cell line Ben-Men 1. Nevertheless, there was no significant difference between grade I and high (II/III) grade meningiomas. Black: WHO grade I, Grey: WHO grade II, White: WHO grade III (PPTX 60 KB)

## Data Availability

Data is not provided.
