# Supplementary figures and images for: Expression of decitabine-targeted oncogenes in meningiomas in vivo
Source: Neurosurg Rev. 2022 Apr 21;45(4):2767–75. doi: 10.1007/s10143-022-01789-1 (PMC9349086; doi:10.1007/s10143-022-01789-1)

## Slide 1
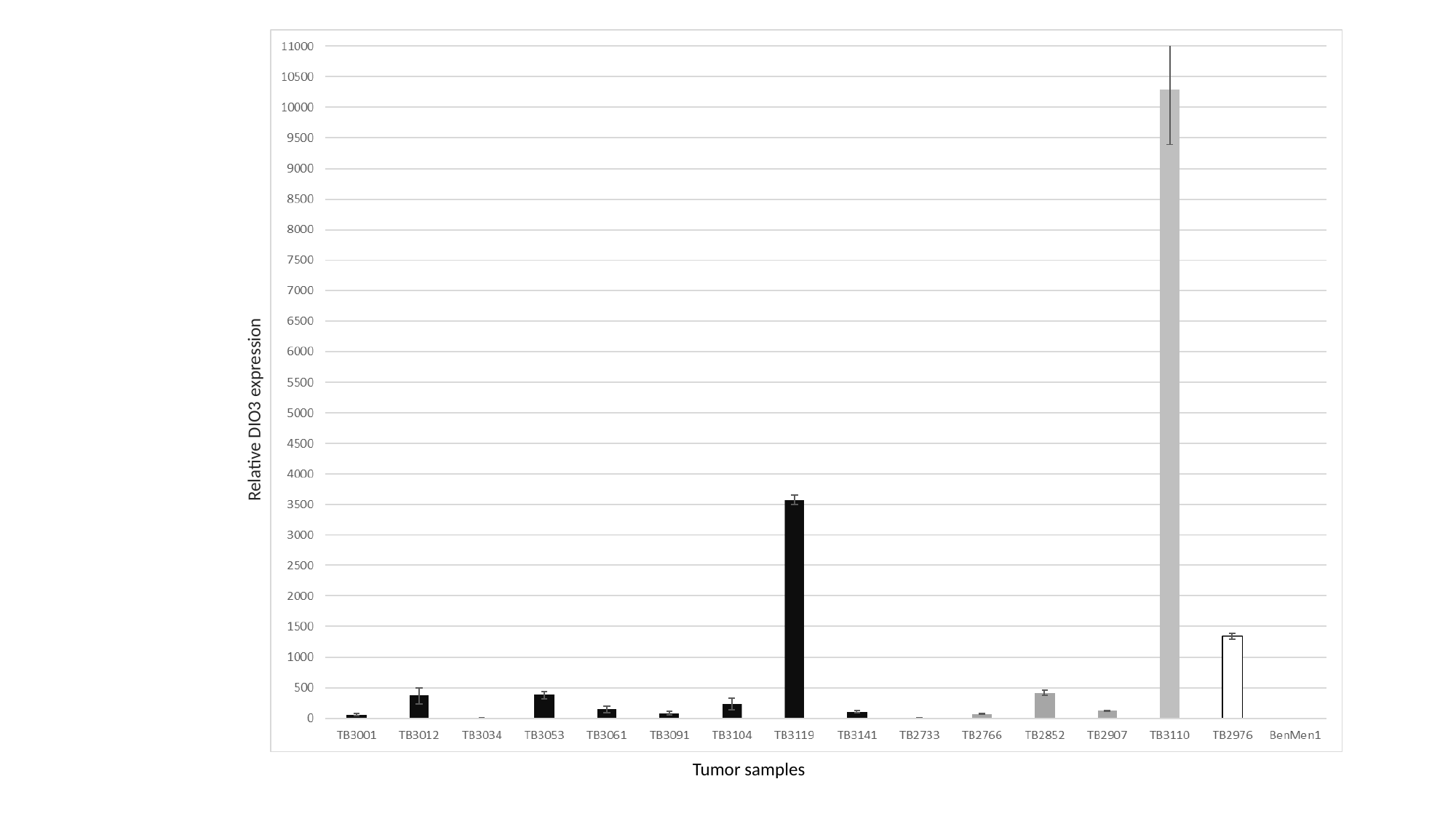

Relative DIO3 expression
Tumor samples

Supplement: Supplementary file 1 — Supplementary file1 Suppl. Fig1 qRT-PCR data of DIO3 expression in meningiomas. DIO3-RNA was expressed in all investigated frozen samples to a distinctly higher level as compared to the DCT-resistant meningioma reference cell line Ben-Men 1. Nevertheless, there was no significant difference between grade I and high (II/III) grade meningiomas. Black: WHO grade I, Grey: WHO grade II, White: WHO grade III (PPTX 60 KB) [file 10143_2022_1789_MOESM1_ESM.pptx]
